# Physiological Factors of Exercise Intolerance in Patients With Pectus Excavatum: A Retrospective, Single-Center Study

**DOI:** 10.7759/cureus.114556

**Published:** 2026-08-14

**Authors:** Roy Chen, Ran Zhang, Bahram Kakavand

**Affiliations:** 1 Department of Medicine, University of South Florida, Tampa, USA; 2 Biostatistics, Biomedical Research Informatics Center, Wilmington, USA; 3 Pediatric Cardiology, Nemours Children's Health, Orlando, USA

**Keywords:** cardiopulmonary exercise testing (cpet), exercise intolerance, haller index, pectus excavatum, vd/vt

## Abstract

Introduction: Pectus excavatum (PE) causes a sunken thoracic wall, which is associated with adolescent exercise intolerance. Since the underlying physiological factors remain uncertain, this retrospective chart review addressed this knowledge gap by reviewing cardiopulmonary exercise tests (CPETs) and identifying physiological factors of exercise intolerance.

Methods: With the Nemours Institutional Review Board’s approval (IRB approval number: STUDY00000806), this study enrolled patients with PE who underwent CPET and chest magnetic resonance imaging (MRI) for Haller index at the Nemours Children’s Hospital, Orlando, Florida, from January 2025 to January 2026. Inclusion criteria included age between 12 and 18 years, Haller index greater than 3.25, and completed CPET. Statistical analysis involved paired-sample parametric tests with Benjamini-Hochberg False Discovery Rate (FDR) adjustment and Spearman’s rank correlation.

Results: Twenty-seven patients (23 male, age 14.9 ± 1.8 years) were enrolled. The mean Haller index was 5.8 ± 3.6. Patients with PE demonstrated significantly lower peak oxygen uptake (VO_2_), oxygen pulse, and anaerobic threshold compared to expected values derived from pediatric ramp-cycle equations. Patients’ average transcutaneous partial pressure of carbon dioxide (P_tc_CO_2_) and dead space to tidal volume ratio (VD/VT) at peak exercise were higher than the normative population values. Oxygen pulse had the strongest inverse association with Haller Index, but this was not statistically significant following FDR adjustments (95% CI: -0.674, -0.018; FDR p = 0.372).

Conclusion: Exercise limitation in patients with PE was corroborated by low peak VO_2_. This could be explained by a significantly lower oxygen pulse at peak exercise. Future studies should examine these findings in prospective trials.

## Introduction

Pectus excavatum (PE) is the most prevalent congenital abnormality of the thoracic wall [1,2,3]. Although its prevalence is 0.4% in the general population, PE accounts for 88% of all congenital chest wall deformities [1,4]. PE is characterized by a concavity of the sternum, ribs, and costal cartilage [1,2]. Its severity is determined by the depth of the concavity, where the deepest point is often located at the xiphisternal joint [1]. PE also exhibits heterogeneity in its shape and symmetry [1,3]. With computed tomography (CT) imaging, these three attributes are graded to help determine the severity of PE [1].

The deformity can lead patients to experience psychological distress, including depression and low self-esteem [1]. PE is also associated with physical symptoms that worsen with age [3]. Patients with PE usually develop a tall and thin physique [2]. When they become adolescents, the flexibility of their thoracic walls decreases [1]. This leads to symptoms of chest pain, shortness of breath, cardiac dysfunction, decreased endurance, and exercise intolerance among adolescents [1,3]. Severe PE can induce diastolic dysfunction through heart displacement, which worsens exercise intolerance [2]. Low cardiopulmonary fitness is associated with higher all-cause mortality [5]. Thus, it is crucial to treat exercise intolerance to help patients maintain fitness.

However, for patients with PE, the underlying physiological factors for exercise intolerance remain uncertain. This knowledge gap complicates assessment of PE’s severity. It also affects patients’ referral to specialists alongside insurance coverage for treatments [6]. To determine the physiological factors of exercise intolerance, cardiopulmonary exercise testing (CPET) has been used to assess exercise response among patients with PE [6]. Through a retrospective chart review, this study aimed to evaluate CPET parameters in adolescents with PE to identify physiological factors associated with exercise intolerance. A secondary objective was to examine whether the severity of chest wall deformity, measured by the Haller Index, correlates with exercise performance.

## Materials and methods

**Figure 1:**
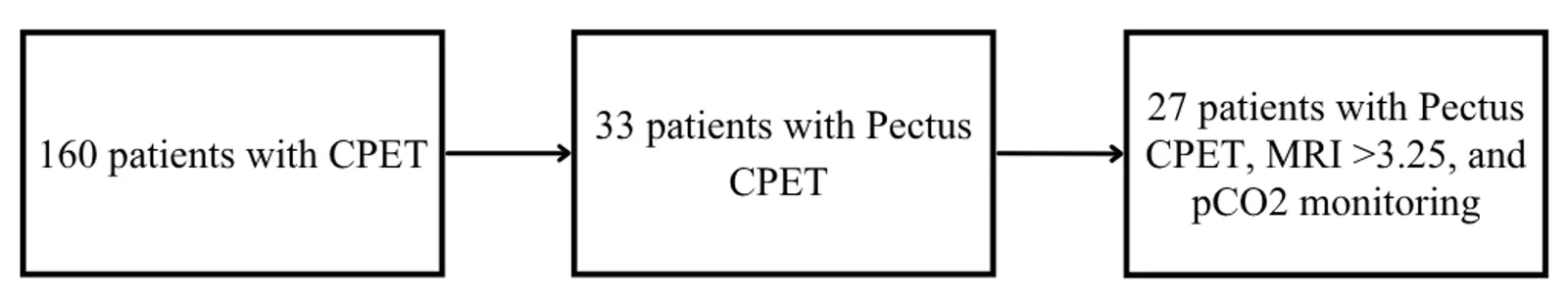
Patient selection This flow chart shows the process of patient selection. Each step includes all eligible patients at the Nemours Children's Hospital in Orlando, Florida, between January 2025 and January 2026.

This study was approved by the Nemours Institutional Review Board (IRB approval number: STUDY00000806). A retrospective chart review was conducted on patients with PE at the Nemours Children's Hospital in Orlando, Florida, between January 2025 and January 2026. Inclusion criteria included age between 12 and 18 years of any sex. Inclusion criteria also included completed CPET and completed chest magnetic resonance imaging (MRI) with a Haller index greater than 3.25 [7]. Patients who lacked the Haller index were excluded from the study. The sample represented all eligible patients. Figure 1 shows the process of patient selection.

All CPETs were performed on a Vyaire Vyntus CPX (Mettawa, IL). Our lab routinely calibrated the CPET equipment before the first patient test and after every two patient tests. CPET was performed after a two-hour fasting period using a 10-15 Watts per minute ramp protocol on a cycle ergometer to exhaustion. Maximal exercise was a symptom-limited test to exhaustion supported by subjective maximal effort of 8-10 as measured on the modified Borg scale (0-10), achieving maximal or near-maximal predicted heart rate, and respiratory quotient >1.1.

Standard variables were collected. These variables included peak oxygen consumption (VO_2_), indexed VO_2_, percent predicted VO_2_, anaerobic threshold (AT), percent predicted AT, oxygen pulse (O_2_P), ventilatory equivalent of CO_2_ production (VE/VCO_2_) slope, and one-minute heart recovery (HRR). AT was determined using the V slope method and confirmed using the VO_2_-VCO_2 _relationship, metabolic equivalent, and end-tidal panels. In addition, HRR was calculated as the difference between the peak heart rate and heart rate recorded one minute after exercise cessation. Heart-rate recovery data were available for 25 of 27 participants. No imputation was performed; analyses of heart-rate recovery were based on participants with available measurements. The investigator analyzing CPET was blinded to the Haller index.

We also measured the oxygen uptake efficiency slope (OUES), its indexed value, and dead space to tidal volume ratio (VD/VT) using the transcutaneous capillary partial pressure of carbon dioxide (P_tc_CO_2_) (TCM Tosca, Radiometer America, Inc.). OUES was derived from VO2 = a log(V_E_) + b [8]. VD/VT was obtained from the modified Bohr equation, which is displayed as Equation 1 [9].

$\frac{VD}{VT} = 1 - \frac{863}{P_{a}CO2 \times \frac{VE}{VCO2}} \tag{1}$

Here, arterial PCO_2_ (P_a_CO_2_) was replaced by transcutaneous PCO_2_ (P_tc_CO_2_), which was shown to closely approximate P_a_CO_2_ [10]. To monitor transcutaneous pCO_2_, a Severinghaus-type sensor was placed on the earlobe. Heating the skin to 42-44 degrees Celsius caused vasodilation with improved capillary blood flow. This allowed diffusion of CO_2_ from the capillaries across the skin into the electrode. CO_2_ caused changes in pH, which is measured and scaled to pCO_2_.

Statistical analysis

Continuous variables were summarized as mean ± standard deviation and median (first and third quartiles). Normal distribution of variables was assessed using the Shapiro-Wilk test. We also evaluated normality using graphical models (histograms, boxplots, and Q-Q plots). Results of CPET variables were compared to published population normative values.

Some CPET variables were compared against individualized expected values derived from validated pediatric ramp-cycle equations [11]. Expected values were calculated using participant age, sex, BMI, and race. Formal comparisons were limited to peak VO_2_, VO_2_ at AT, VO_2_ at AT to peak VO_2_ ratio, peak work rate, peak O_2_P, and VE/VCO_2_ slope, which were directly covered by the equations. Paired-sample parametric tests were administered after the normality of the datasets was confirmed. Outcomes without directly applicable equations were compared to published population normative values without adjustment for body size or sex.

Because multiple CPET variables were compared against published reference values, we applied Benjamini-Hochberg false discovery rate (FDR) adjustment [12]. Lastly, we compared CPET variables with the Haller index using Spearman rank correlation to assess any association between pectus severity and exercise performance. Fisher’s z-transformation was used to determine each Spearman coefficient’s 95% confidence interval.

## Results

This study included 27 adolescents with PE who had an average age of 14.89 years. The mean MRI-pectus time difference was 40.1 days, with a standard deviation of 37.2 days (0-149 days). The descriptive statistics of the patients’ characteristics are shown in Table 1.

**Table 1:** Patient characteristics n, number of patients; SD, standard deviation; Q1, first quartile; Q3, third quartile

Variable	N	Mean ± SD	Median (Q1, Q3)	Range
Age (years)	27	14.89 ± 1.76	15.00 (14.00, 16.50)	6
Weight (kg)	27	55.56 ± 7.56	55.60 (50.55, 61.35)	34.7
Height (m)	27	1.73 ± 0.09	1.73 (1.70, 1.79)	0.35
BMI (kg/m²)	27	18.54 ± 2.44	18.45 (16.79, 20.12)	10.31
Haller index	27	5.82 ± 3.58	4.60 (4.07, 6.70)	18.63
Male, n (%)	23 (85.2%)			
Female, n (%)	4 (14.8%)			
Caucasian, n (%)	27 (100%)			

All variables were normally distributed except for the Haller index, which was mainly due to a patient with an outlier value of 21.25. We confirmed the accuracy of this value and elected to include this patient in the final analysis.

Compared to expected values of healthy adolescents, patients with PE demonstrated significant limitations in exercise performance. This is shown in Table 2, where all of its variables meet the assumptions for a paired-sample parametric test due to the normality of their datasets. Among patients with PE, these CPET variables demonstrated statistically significant differences from the expected values even after Benjamini-Hochberg multiplicity adjustment.

**Table 2:** CPET outcomes compared with individualized pediatric expected values Expected values were calculated individually from the sex-specific Burstein et al. pediatric ramp-cycle equations using age, BMI, and race (all participants were White) [11]. † Mean difference = observed minus expected. P-values are from one-sample t-tests of the individual observed-minus-expected differences; FDR adjustment was applied across the six outcomes. CPET, cardiopulmonary exercise testing

Variable	Observed mean ± SD	Expected mean ± SD*	Mean difference (95% CI)†	P-value^2^	FDR-adjusted P-value^3^
Peak VO₂ (L/min)	1.57 ± 0.36	2.48 ± 0.40	-0.91 (-1.07 to -0.75)	9.26 × 10⁻¹²	3.70 × 10⁻¹¹
VO₂ at AT (L/min)	0.92 ± 0.27	1.37 ± 0.21	-0.44 (-0.56 to -0.33)	2.00 × 10⁻⁸	4.00 × 10⁻⁸
VO₂ at AT / peak VO₂ (%)	37.7 ± 11.2	55.9 ± 1.6	-18.2 (-22.7 to -13.8)	6.26 × 10⁻⁹	1.67 × 10⁻⁸
Peak work rate (W)	111.0 ± 29.7	198.6 ± 32.5	-87.6 (-100.2 to -75.1)	7.08 × 10⁻¹⁴	5.67 × 10⁻¹³
Peak O₂ pulse (mL)	9.0 ± 2.1	12.2 ± 3.4	-3.3 (-4.4 to -2.1)	3.58 × 10⁻⁶	5.73 × 10⁻⁶
VE/VCO₂ slope	27.3 ± 2.6	28.7 ± 1.0	-1.4 (-2.4 to -0.3)	1.19 × 10⁻²	1.19 × 10⁻²

For variables without directly applicable equations, they were compared to published normative population values. This included variables related to exercise capacity and cardiovascular response (Table 3). Although two patients lacked HRR values, descriptive statistics were computed based on the available participant data.

**Table 3:** Comparison of exercise capacity and cardiovascular response variables with published reference values n, number of patients; SD, standard deviation; Q1, first quartile; Q3, third quartile; VO2, peak oxygen consumption; AT, anaerobic threshold; HRR, one-minute heart rate recovery; WR, work rate; O2P, oxygen pulse

Variable	Normative value	Reference	n	Mean ± SD	Median (Q1, Q3)
% predicted VO_2_	85%	[13]	27	64% ± 14%	64% (54%, 75%)
VO_2_/kg (mL/min/kg)	38.6	[14]	27	28.5 ± 7.2	28.7 (23.7, 33.9)
% predicted VO_2_/kg	85%	[13]	27	64% ± 14%	63% (54%, 75%)
% AT	40%	[15]	27	38% ± 11%	39% (32%, 43%)
HRR (bpm)	18	[16]	25	28 ± 11	28 (21, 33)
VO_2_/WR (mL/min/W)	10.3	[17]	27	9.7 ± 1.6	9.7 (8.6, 10.9)
% O_2_P	80%	[13]	27	71% ± 14%	69% (62%, 80%)

In addition, variables related to gas exchange and ventilatory efficiency were compared to published normative population values (Table 4). The average peak VD/VT was higher than the normative value, while the average OUES and indexed OUES were lower compared to the general population’s normative values. This suggested lower aerobic exercise capacity.

**Table 4:** Comparison of gas exchange and ventilatory efficiency variables with published reference values n, number of patients; SD, standard deviation; Q1, first quartile; Q3, third quartile; OUES, oxygen uptake efficiency slope; OUESi, oxygen uptake efficiency slope index; PCO2, partial pressure of carbon dioxide; VD/VT, dead space to tidal volume ratio

Variable	Normative value	Reference	n	Mean ± SD	Median (Q1, Q3)
OUES (L/min)	1.98	[18]	27	1.77 ± 0.37	1.78 (1.52, 1.92)
OUESi (mL/kg/min)	38.4	[19]	27	32.1 ± 6.3	33.2 (28.1, 36.1)
Base PCO_2 _(mmHg)	40	[20]	27	42.2 ± 2.8	42.0 (40.5, 43.5)
Peak PCO_2 _(mmHg)	38	[21]	27	43.7 ± 3.0	43.0 (42.0, 46.0)
Rest VD/VT (%)	30%	[22]	27	33% ± 7%	35% (27%, 38%)
Peak VD/VT (%)	17%	[23]	27	21% ± 5%	21% (19%, 24%)

Spearman rank correlation studies revealed no statistically significant associations between Haller index and CPET variables after adjustment for multiple comparisons (Table 5). Oxygen pulse demonstrated the strongest inverse association with Haller Index in the unadjusted analysis; however, this association was not statistically significant after FDR adjustment (ρ = −0.395; unadjusted p = 0.041; FDR-adjusted p = 0.372). A sensitivity analysis excluded the patient with a Haller index of 21.25. It produced similar correlation estimates that did not change the overall conclusions.

**Table 5:** Spearman’s rank correlation coefficients between the Haller index and CPET variables CPET, cardiopulmonary exercise testing; ρ, Spearman’s rank correlation coefficient; CI, confidence interval; FDR, false discovery rate; VO_2_, peak oxygen consumption; AT, anaerobic threshold; VE/VCO_2_, ventilatory equivalent of CO_2_ production; VD/VT, dead space to tidal volume ratio; OUES, oxygen uptake efficiency slope; OUESi, oxygen uptake efficiency slope index; O_2_P, oxygen pulse

Variable	ρ	95% CI	P-value	FDR P-value
Peak VO_2_ (L/min)	-0.301	(-0.611, 0.090)	0.127	0.382
AT (L/min)	-0.334	(-0.634, 0.053)	0.088	0.382
% AT	-0.186	(-0.529, 0.209)	0.352	0.553
VE/VCO_2_ slope	0.248	(-0.146, 0.574)	0.212	0.477
Rest VD/VT (%)	-0.059	(-0.429, 0.329)	0.771	0.794
Peak VD/VT (%)	0.067	(-0.322, 0.436)	0.741	0.794
OUES (L/min)	-0.180	(-0.524, 0.215)	0.369	0.553
OUESi (mL/kg/min)	-0.053	(-0.424, 0.334)	0.794	0.794
O_2_P (mL)	-0.395	(-0.674, -0.018)	0.041	0.372

## Discussion

In patients with PE, the sternum may compress against the right side of the heart, which can limit maximal right ventricular filling [24]. When patients with PE exercise, the decreased ventricular output may lead to tachycardia, which increases the rate of fatigue [24]. To assess this mechanism, cardiac MRI is used to assess the Haller index, which indicates the severity of PE [7]. In addition, CPET is done to elucidate the physiological factors of exercise intolerance in patients with PE [6].

In this retrospective study of adolescents with PE, patients demonstrated lower aerobic capacity as indicated by the significantly lower peak VO_2_. Patients with PE also had lower average OUES compared to healthy adolescents. Although OUES lacked a formal p-value, it has the advantage of utility in submaximal CPET [25]. An important finding was significantly reduced peak O_2_P among patients with PE. Low O_2_P may indicate lower stroke volume [24]. It may also indicate lower oxygen extraction by the peripheral muscles [24]. These mechanisms may explain the low exercise performance of patients with PE.

In contrast to Oleksak et al.’s findings, the mean VE/VCO2 slope in our patient population was statistically lower than the published values [26]. We explain this discrepancy through another finding of this study: the higher peak transcutaneous PCO_2_ in patients with PE. This can be explained by rearrangement of the above-modified Bohr equation, as shown in Equation 2 [9].

$\frac{VE}{VCO2} = \frac{863}{P_{tc}CO2 \times (1 - \frac{VD}{VT})} \tag{2}$

According to the modified Bohr equation (Equation 2), decreased VE/VCO_2 _may be influenced by a decrease in VD/VT and/or an increase in P_tc_CO2 [9]. We believe that the balance between elevated peak VD/VT and increased PCO_2_ in our patients can potentially result in a VE/VCO2 slope that is lower than the expected value.

To evaluate whether pectus severity was associated with exercise performance, we examined the Haller index against selected CPET variables for correlation using Spearman rank correlation. No statistically significant associations were found after adjustment for multiple comparisons. Thus, patients with PE show impaired exercise capacity, but the severity of anatomical deformity was not clearly associated with CPET impairment in this sample.

This study’s results were clinically significant. They provided evidence for the use of oxygen pulse as an indication for surgery. Specifically, the Nuss repair has been shown to significantly improve peak oxygen uptake and oxygen pulse for patients with PE [27]. One study attributes this procedure's improvement in exercise capacity to increased oxygen pulse, which indicates improved stroke volume [27]. Given that oxygen pulse is derived from CPET, this study supports the use of CPET for establishing surgical indications in patient management of pectus excavatum.

Several limitations should be considered for this study. As a single-center analysis with a relatively small sample of 27 patients, this study had limited external validity. The small sample size limited the precision of the correlation estimates and the ability to detect modest associations between the Haller Index and CPET variables. Therefore, non-significant findings should not be interpreted as evidence of no association. This study should be considered exploratory and hypothesis-generating. In addition, due to its retrospective nature, this study was vulnerable to selection bias. Namely, only patients referred for both MRI and CPET were included. These individuals may represent more symptomatic cases of PE, so they may not be representative of the broader patient population with PE. Heart-rate recovery data were missing for two participants. Although the proportion of missing data was small, the potential for bias cannot be excluded if missingness was related to exercise performance, test completion, or post-exercise measurement procedures.

This study cannot establish causation due to its observational design. This study also lacked a control group, so significance tests were conducted using expected values derived from validated pediatric ramp-cycle equations. Although the comparisons were matched for age, sex, BMI, and race, it cannot control for differences in exercise protocol, equipment, and study protocols. Thus, the use of predicted values introduces heterogeneity, decreases internal validity, and prevents causal interpretation. Potential confounding factors include habitual physical activity, sports participation, pulmonary function, respiratory comorbidities, connective-tissue disorders, and prior thoracic procedures. These factors were not consistently available in the retrospective records. Consequently, the observed CPET findings cannot be attributed solely to pectus excavatum or the Haller index. Future prospective studies should examine oxygen pulse's relationship with exercise intolerance in patients with PE.

## Conclusions

This retrospective study reviewed the charts of 27 adolescents to identify the physiological factors for exercise intolerance in patients with PE. Reduced O_2_P may contribute to, or may be associated with, exercise limitation. This warrants further investigation in prospective controlled studies with matched controls, exercise cardiac assessments, and exercise pulmonary assessments.
